# Assessing Abundance and Species Composition of Thrips (Thysanoptera) in Florida Lettuce Fields and Optimizing Monitoring Methods

**DOI:** 10.3390/insects17070676

**Published:** 2026-06-28

**Authors:** Yavonne E. Williams, Tennyson Bilinkhinyu Nkhoma, Felipe N. Soto-Adames, Laura V. Bautista-Romero, Germán V. Sandoya, De-Fen Mou

**Affiliations:** 1 Department of Entomology and Nematology, Everglades Research and Education Center, University of Florida, Belle Glade, FL 33430, USA; 2 Division of Plant Industry, Florida Department of Agriculture and Consumer Services, Gainesville, FL 32608, USA; 3 Horticultural Science Department, Everglades Research and Education Center, University of Florida, Belle Glade, FL 33430, USA

**Keywords:** insect vectors, insect, virus transmission, orthotospovirus, leafy vegetables

## Abstract

*Frankliniella occidentalis* is a major threat to lettuce production due to its role in transmitting *Impatiens necrotic spot virus* (INSV). To better understand the potential risk of INSV outbreaks in Florida lettuce production areas, this study aimed to assess thrips population abundance and species composition. Five methods, whole plant sampling, yellow sticky traps, blue sticky traps, yellow pan traps, and blue pan traps, were evaluated for thrips collection. Traps were placed in one research and two commercial lettuce farms for two growth cycles, Fall 2023 and Spring 2024. Sticky traps collected the highest number of thrips followed by pan traps, and whole plant sampling. Although sticky traps collected the highest number of thrips, specimens were in poor condition and not easily identifiable to species, which is an important step in determining the risk of INSV outbreaks. In contrast, pan traps collected the second highest number of thrips, and preserved specimens well for later identification. Overall, at least eleven species were identified using these sampling methods, the abundant species were *Frankliniella bispinosa*, *Microcephalothrips abdominalis*, and *Leucothrips piercei*. Vectors of orthotospoviruses, *F. occidentalis*, *F. fusca*, and *F. schultzei*, were also detected, underscoring the importance of continued thrips surveillance to safeguard Florida lettuce from thrips and thrips-transmitted viruses.

## Simple Summary

Thrips are widely distributed pests that affect many plant species, including vegetable and ornamental crops. Some thrips species can also transmit plant viruses, leading to additional significant economic losses. Specifically, thrips-transmitted orthotospoviruses are major threats to lettuce production in California, and there is concern that similar problem could occur in southern Florida, the third-largest lettuce producing region in the United States. To better understand the prevalence of thrips pests and thrips-transmitted viruses, this study characterized thrips species and population levels present in lettuce in southern Florida. Furthermore, this study compared different sampling methods, including whole plant sampling, blue and yellow pan traps, blue and yellow sticky traps, for thrips population monitoring in lettuce. Whole plant sampling yielded the lowest thrips counts, while sticky traps captured the most. However, pan traps were more practical, due to their effectiveness in capturing thrips and preserving specimens for species identification. The most abundant species was the Florida flower thrips (*Frankliniella bispinosa*), which is not currently considered a pest of lettuce. However, important vectors of orthotospoviruses, such as the Western flower thrips (*Frankliniella occidentalis*), were detected. This highlights the importance of thrips surveillance for lettuce crop protection.

## 1. Introduction

Many species of thrips, particularly those species belonging to the family Thripidae within the order Thysanoptera, cause direct physical damage to plants by feeding on leaves, flowers, and fruits. Their feeding behavior involves extracting cell contents, resulting in wrinkled and silvery lesions that develop to necrosis, as well as deformed and scarred leaves and fruits [1,2]. The feeding damage negatively affects photosynthetic activity and overall plant health and yield. In addition to direct physical damage, certain thrips species act as vectors for plant viruses, causing significant economic losses in agriculture. To date, fifteen thrips species have been identified as vectors of orthotospoviruses [3,4].

Among the thrips vectors, *Frankliniella occidentalis* (Pergande), the Western flower thrips, is a prominent pest and a major vector of *Impatiens necrotic spot virus* (INSV) [5]. The virus belongs to the genus *Orthotospovirus* and family Tospoviridae, and affects a wide range of crops, including lettuce (*Lactuca sativa*). *Impatiens necrotic spot virus* was first detected in lettuce in California in 2006 [6] and has become a major limiting factor in production since 2019, with up to 100% yield losses [7]. Recently, INSV has also been reported in Arizona and Mexico [8,9]. Symptoms of INSV in lettuce include necrotic spots, chlorosis on leaves, and browning of the midrib, all of which impair plant growth and results in unmarketable lettuce heads [7,10].

Florida is the third largest lettuce-producing state in the U.S., after California and Arizona. Lettuce production primarily located in the Everglades Agricultural Area (EAA) in Florida, where approximately 47 km^2^ (11,713 acres) are cultivated [11]. Although INSV has not been reported in Florida in lettuce, its potential introduction and establishment remain a concern for growers. This is due to the possible introduction of viruliferous thrips through commercial trade of lettuce or other produce from affected regions. In addition, INSV has previously been reported in Florida in ornamental plants, including lisianthus (*Eustoma grandiflorum*) and *Phalaenopsis* orchids [12,13]. However, whether INSV is widely spread beyond these plants in Florida is unknown. Considering the risk of viruliferous thrips incursion, the historical incidences of INSV in the state, and the prevalence of thrips pests in Florida’s agricultural landscape [14,15,16], there is an urgent need to better understand the potential impact of thrips and thrips-transmitted viruses on lettuce production in the EAA.

Effective monitoring to assess the presence of the target pest is a critical step in integrated pest management. However, scouting for thrips on plants is particularly challenging due to their small size and cryptic behaviors, such as residing in concealed parts of the plants [17]. Various scouting methods can be used to monitor thrips populations, including flower sampling, leaf sampling, beating, sweep nets, suction traps, sticky traps, and pan traps [18]. However, the efficacy of these methods in field conditions can vary depending on various factors, such as crop surveyed, environmental conditions, trap color, placement strategy, and the use of attractants [19]. Thrips scouting in lettuce presents unique challenges due to the crop’s dense canopy, the fragile leaves that are unsuitable for sweep net or beating, and the absence of flowers as sampling tissue in commercial production. Despite the existence of various thrips scouting methods, there is limited information on evaluating their effectiveness in lettuce cropping systems.

This study evaluated different scouting methods to determine their efficacy in monitoring thrips populations in lettuce. Whole plant sampling, blue pan trap, yellow pan trap, blue sticky trap, and yellow sticky trapwere assessed under field conditions. Additionally, since not all thrips species cause the same level of damage or are competent vectors of viruses, species identification was conducted when possible, to compare the species composition collected by different methods. This study aimed to support lettuce production in Florida by providing information on thrips population abundance and the species present in the area, along with suitable scouting methods for thrips surveillance.

## 2. Materials and Methods

### 2.1. Field Sites

**Figure 1:**
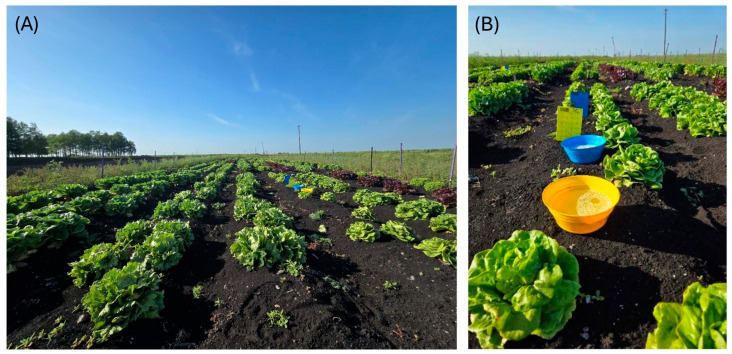
Sampling plot on the research farm: (**A**) Wide shot of plot with pan traps and sticky traps placed between lettuce. (**B**) Close up of pan traps and sticky traps.

Field experiments were conducted at the research farm at the Everglades Research and Education Center (EREC), University of Florida in Belle Glade, Florida, as well as two commercial plots in a commercial farm in Belle Glade, Florida. Two different plots, R1 and R2, approximately 2 km apart, were used in Fall 2023 (R1) and Spring 2024 (R2) on the research farm due to area limitation. Plot R1 (Fall 2023) was planted on 26 October 2023, and the plot R2 (Spring 2024) was planted on 1 February 2024. Each plot was approximately 10 m wide, and 30 m long, consisting of eight beds with 2 rows of lettuce planted 46 cm apart (Figure 1). Eight lettuce cultivars were planted in a randomized complete block design with 4 blocks of each cultivar. Lettuces were planted from seeds using hand planter, and the seedings were manually thinned after germination to maintain approximately 20 cm spacing between plants. No pesticides were applied during the experiments. The entire EREC research farm covers approximately 3 km^2^, with most of the land planted in sugarcane and smaller sections planted in lettuce, celery, corn, and natural weeds.

Additionally, C1 and C2 in Fall 2023 as well as C3 and C4 in Spring 2024, were surveyed at a commercial farm located in the EAA, Belle Glade, Florida. Plots C1 and C2 in the commercial field were planted on 7 October 2023, and 4 October 2023, respectively, and were considered the Fall 2023 season. Plots C3 and C4 were planted on 31 December 2023, and 1 December 2023, respectively, and were considered the Spring 2024 season. Within each plot, approximately 30 m by 30 m was designated as the experimental area for setting up traps, within a plot size of approximately 180 m by 350 m. The beds in the commercial farms were planted with four rows of lettuce approximately 46 cm apart. The commercial fields received pesticides applications as part of the grower’s standard commercial production procedures. Due to the discretion of the growers, the trade names and active ingredients of the pesticides used were not made available.

### 2.2. Evaluation of Sampling Method for Thrips

Samples were collected weekly in both research and commercial farms. Five sampling methods—whole plant sampling, blue pan trap, yellow pan trap, blue sticky trap, and yellow sticky traps were assessed in the research field; while only four methods—blue pan trap, yellow pan trap, blue sticky trap, and yellow sticky traps were evaluated in the commercial field.

#### 2.2.1. Whole Plant Samples

To assess thrips abundance and species from the lettuce plants, one randomly selected lettuce plant was collected from each cultivar and immediately placed in a plastic sealable bag (33 cm × 38.1 cm, Ziploc 2 Gallon, SC Johnson, Racine, WI, USA) filled with 90% ethanol. The collected samples were then kept in the sealable bags until further processing in the laboratory. Lettuce sample collection was conducted only in the research field, and sampling began five weeks after planting in both the Fall 2023 and the Spring 2024 experiments.

In the laboratory, lettuce plants were agitated in collection bags to dislodge thrips. Leaves were then separated with forceps, rinsed thoroughly in a 1 L collection beaker, and discarded. The content from the beaker was filtered through a strainer assembled from an embroidery hoop (10 cm diameter) fitted with fine mesh (200 mm Solid Chiffon Fabric, Casa Collection, Brooklyn, NY, USA). After all leaves were removed from the collection bag, the remaining ethanol was poured through the same strainer. The strainer, now containing all the filtered contents, was then inverted over a Petri dish (150 mm diameter with 15 mm height) and rinsed with 50% ethanol ensuring that the entire surface area of the mesh was washed, and all thrips specimens were ejected. The mesh was washed after every sample to ensure no cross contamination between samples. The Petri dishes containing thrips specimens were examined under a dissecting microscope (Leica S APO, Wetzlar, Germany) for thrips counting and species identification (see Section 2.2.4).

#### 2.2.2. Pan Trap Samples

Pan traps were made using yellow and blue colored 1.8 L snack bowls (20 cm opening diameter × 12 cm height, Mintra USA, Marlborough, MA, USA) and filled up to 60% of their capacity with a pan trap solution composed of 80% propylene glycol mixed with 20% water, a scoop of rock salt, and a few drops of dishwashing soap (Dawn) per 3.79 L. Pan traps were positioned on the ground throughout the survey period. Pan traps were slightly higher than the lettuce canopy during early growth stages and approximately at canopy height during later growth stages (Figure 1). In the research farm, three blue and three yellow pan traps were placed throughout the field. In the commercial fields, a total of 8 pan traps, four pan traps of each color, were placed at each sampling plot. The pan trap samples (i.e., the solution containing thrips) were collected by transferring the solutions to a 1 L plastic deli containers (Heavy-Duty Deli Containers S-22771, 11 cm diameter and 14 cm height, ULINE, Pleasant Prairie, WI, USA) and rinsing the bowl with water into the collection container to ensure all contents were collected. The pan traps were collected and refilled weekly. In the laboratory, pan trap samples in the deli containers were filtered using the same strainer as used in the whole plant sample processing, and the deli container was rinsed with water to rinse out any content left behind. The specimens were then transferred to a Petri dish and counted under a microscope. Given the high volume of samples and number of thrips collected, a maximum of randomly selected 15 thrips were identified per sample.

#### 2.2.3. Sticky Traps

Three blue and three yellow sticky traps (Arbico organics, Oro Valley, AZ, USA) were placed in the research field (Figure 1), and four blue and four yellow traps were placed per sampling plot in the commercial fields. Sticky traps were set up by stapling them to wooden stakes (30 cm length and 3 cm width) and inserting the stakes into the soil, they were higher than the lettuce canopy during early growth stages and approximately at canopy height during later growth stages (Figure 1). Sticky traps were collected and replaced weekly. When collecting, sticky traps were wrapped in plastic wrap to avoid any contamination between traps and placed in a labeled, dated sealable collection bag onsite. When returned to the lab, bags were placed in the freezer to retain specimen quality until processed. Wrapped sticky traps were inspected under a microscope to count the thrips. Due to the low integrity of the samples and difficulty of removing specimens from glue on traps, thrips counted on sticky traps were not identified.

#### 2.2.4. Thrips Identification

All female thrips collected from the whole plant samples and the subset of up to 15 specimens from each pan trap sample were identified under a microscope based on the dichotomous keys of Cleuver & Smith [20] and Hoddle et al. [21]. Those that could not be easily distinguished were slide mounted and keyed to either genus or species level based on *Thysanoptera: An Identification Guide* [22] and *Thrips of California* [21]. All specimens for taxa with 10 or fewer individuals, and a subset of 20% for taxa with more than 10 individuals, were sent to the Florida Department of Agriculture and Consumer Services (FDACS), Gainesville, FL to confirm identification. Voucher specimens were deposited in the Florida Collection of Arthropods at the FDACS.

### 2.3. Data Analysis

Data analyses were conducted in R version 4.2.2 [23]. Firstly, generalized linear models (GLMs) with a negative binomial distribution were fitted using glm.nb function from the MASS package [24] to analyze the number of thrips. In these models, data from each field were analyzed independently, the number of thrips were response variable, sampling method and plant age were fixed effects. Then, analysis of deviance table (Type II Wald Chi-square tests) was performed using “Anova” function from “car” package [25]. The plant age (i.e., weeks after planting) significantly affected the number of thrips for all research and commercial data, and the interaction between sampling method and plant age was significant in research data (Table 1). Therefore, the number of adult thrips collected by different sampling methods were further analyzed separately for each week, and data from each field were analyzed independently.

To evaluate the effects of different sampling methods on the number of thrips captured, GLMs with a negative binomial distribution with log link function were fitted using glm.nb function from the MASS package [24]. In each GLM, the sampling method was treated as a fixed effect (five levels for research fields: whole plant sample, blue pan traps, yellow pan trap, blue sticky trap, and yellow sticky trap; four levels for commercial fields: blue pan trap, yellow pan trap, blue sticky trap, and yellow sticky trap), and the number of adult thrips was the response variable. Estimated marginal means were computed on the log scale and back-transformed to the response scale for interpretation and corresponding 95% confidence intervals were calculated. Pairwise comparisons with Bonferroni correction were performed using the “emmeans” function from the “emmeans” package [26]. Finally, to improve interpretability, “cld” function from the “multcomp” package [27] was used to generate compact letter displays indicating significant differences among sampling methods.

In addition, effects of sampling method on thrips assemblages were tested using permutational multivariate analysis of variance (PERMANOVA) using “adonis2” function from the vegan package [28]. Because only 15 specimens were randomly selected for species identification when more than 15 thrips per sample were counted in pan trap samples, identification rates differed among sampling methods. Therefore, species abundance data may underestimate the occurrence of rare species in some methods. To account for the differences in sample size and uneven identification rates among sampling methods, Hellinger-transformation [29] was applied using the “decostand” function to the raw thrips abundance matrix before distance computation. Bray–Curtis dissimilarities [30] were then used from the transformed data. The number of identified specimens per sample (i.e., identification effort) was included as a covariate in the PERMANOVA analysis to account for variation in identification rates. The PERMANOVA model was community_matrix ~ identification effort + sampling method. Samples with rare species (occurring in fewer than five samples) were removed to reduce noise. Additionally, differences in multivariate dispersion among sampling methods were assessed using the “betadisper” function in the “vegan” package [28]. Patterns in community composition were visualized using non-metric multidimensional scaling (NMDS) [31,32] using the “metaMDS” function from the “vegan” package [28].

## 3. Results

### 3.1. Evaluation of Sampling Method for Thrips Abundance

**Figure 2:**
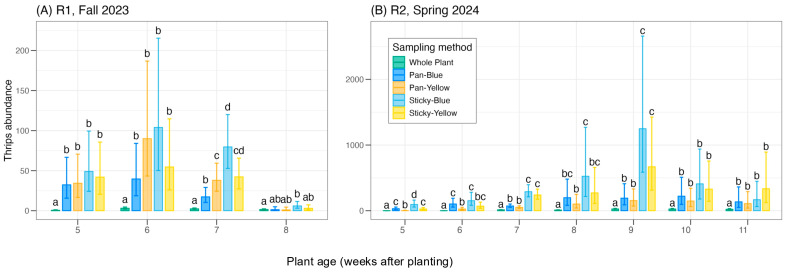
Thrips collected weekly using five sampling methods in lettuce fields at the research farms at the University of Florida Everglades Research and Education Center, Belle Glade, Florida. Bars indicate model estimated thrips abundance with ±95% confident interval. Number of thrips were compared between sampling methods, each week was compared separately. Different letters above the error bars indicate values that are significantly different from each other (*p* < 0.05, Bonferroni test). (**A**) Lettuce planted on 26 October 2023 (considered Fall growing season). (**B**) Lettuce planted on 1 February 2024 (considered Spring growing season).

**Figure 3:**
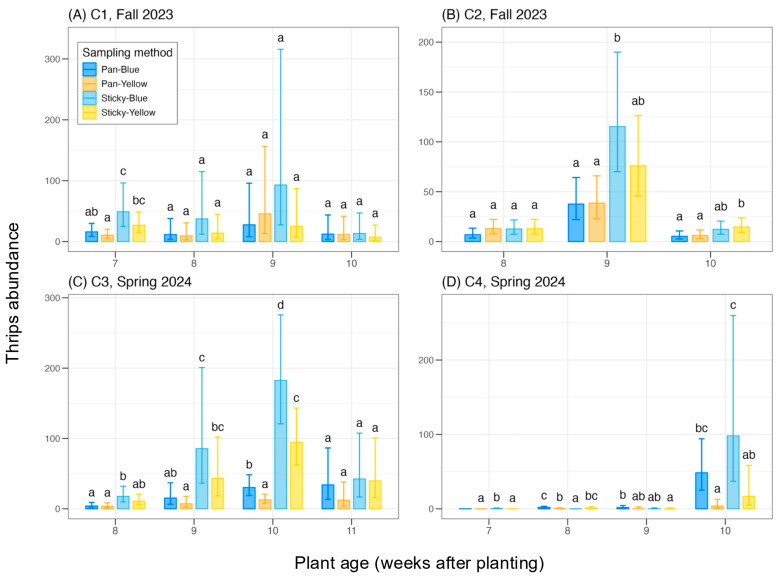
Thrips collected weekly using four sampling methods in four commercial lettuce fields at the Everglades agricultural area, Belle Glade, Florida. Bars indicate model estimated thrips abundance with ± 95% confident interval. Number of thrips were compared between sampling methods, each week was compared separately. Different letters above the error bars indicate values that are significantly different from each other (*p* < 0.05, Bonferroni test). (**A**) Lettuce planted on 7 October 2023 (considered Fall growing season). (**B**) Lettuce planted on 4 October 2023 (considered Fall growing season). (**C**) Lettuce planted on 31 December 2023 (considered Spring growing season). (**D**) Lettuce planted on 1 December 2023 (considered Spring growing season).

The number of thrips captured varied depending on the sampling methods and plant age (Figure 2 and Figure 3). Whole plant samples consistently captured significantly lower number of thrips than other methods, with a mean of 1.9 ± 0.18 (mean ± SE) and a maximum of 10 thrips adults in Fall 2023 (R1), and a mean of 13.99 ± 1.26 and a maximum of 102 thrips adults in Spring 2024 (R2) collected per week (Figure 2).

The difference in thrips numbers among other four methods—blue pan trap, yellow pan trap, blue sticky trap, and yellow sticky trap—were inconsistent across all the experiments conducted in this research (Figure 2 and Figure 3). For example, in the research farm during Fall 2023, no significant difference was observed among the pan and sticky trap methods at weeks 5 and 6. However, at week 7, blue sticky traps captured approximately 2.1 times more thrips than yellow pan traps (z = −3.15, *p* = 0.0165) and 4.6 times more than blue pan traps (z = −5.59, *p* < 0.0001) (Figure 2A). Similarly, at week 5 in Spring 2024, significantly more thrips were collected from blue sticky traps than blue pan traps (z = −3.63, *p* = 0.0028), yellow pan traps (z = −7.84, *p* < 0.0001), and yellow sticky traps (z = 4.08, *p* = 0.0005) (Figure 2B). Blue sticky traps also captured significantly more thrips than yellow pan traps at weeks 6 (z = −5.01, *p* < 0.0001), 7 (z = −9.18, *p* < 0.0001), 8 (z = −3.36, *p* = 0.0077), and 9 (z = −5.01, *p* < 0.0001) (Figure 2A). When comparing blue and yellow pan traps, blue traps captured significantly more thrips at week 5 (z = 5.315, *p* < 0.0001) and week 6 (z = 3.864, *p* = 0.0011) in Spring 2024 (Figure 2).

The inconsistency in thrips captured among the blue and yellow pan and sticky traps was similar in the commercial farms (Figure 3). At week 7 in C1, blue sticky traps captured approximately three times more thrips than blue pan traps (z = −3.032, *p* = 0.0146) and 4.7 times more than yellow pan traps (z = −4.051, *p* = 0.0003) (Figure 3A). Similarly, significant more thrips were captured by blue sticky traps than blue (z = −3.82, *p* = 0.0008) and yellow pan traps (z = −3.735, *p* = 0.0011) at week 9 in C2 (Figure 3B), at week 8, 9, and 10 in C3 (Figure 3C). Blue pan traps collected more thrips than yellow pan traps at week 10 in C3 (z = −3.202, *p* = 0.0082) (Figure 3C), and week 10 in C4 (z = −4.698, *p* < 0.0001) (Figure 3D).

Notably, a higher number of thrips were captured in Spring 2024 than Fall 2023 in the research farms (Figure 2). Across sampling methods and plant ages, in research farms, 14.5 thrips were collected in Fall 2023, and 86.2 adults collected in Spring 2024 per week with a maximum of 1268 thrips collected by the blue sticky trap at week 9. In commercial farms, mean number of thrips collected were similar across the fields and seasons (Figure 3).

### 3.2. Thrips Species Composition

A total of 2502 thrips were captured from the whole plant samples (Table 2). Of these, 2113 specimens (84% of the total captured) belonged to the suborder Terebrantia within the order Thysanoptera and were identified to genus or species level. Eight specimens belonged to the suborder Tubulifera were not further identified to genus or species levels since they are commonly mycophagous and not considered agricultural pests. Other unidentified specimens were either males or too damaged to observe their morphological characters needed for genus or species level identification. In research fields, a total of 2273 and 3131 thrips were collected using yellow and blue pan traps, respectively, whereas in commercial fields, a total of 650 and 1258 thrips were collected in yellow and blue pan traps, respectively. Approximately 13.3% to 62% specimens collected from pan traps were identified to genus or species level, mainly due to limited personnel (Table 2). Thrips counted on sticky traps were not identified because of the low integrity of the samples and difficulty of removing specimens from glue on traps.

**Figure 4:**
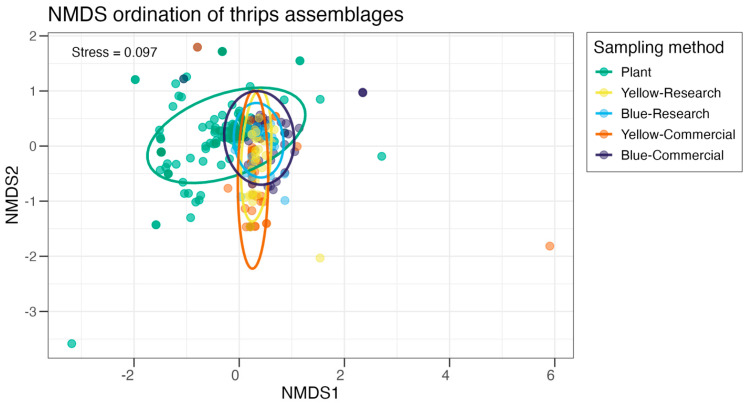
NMDS ordination of thrips assemblages by sampling method based on Bray–Curtis dissimilarities (stress = 0.097). Points represent samples and colors indicate methods; ellipses show 95% confidence regions. Partial separation among methods and differences in dispersion are evident.

Thrips species composition differed significantly among sampling methods (PERMANOVA, Bray–Curtis dissimilarity, 999 permutations; F = 22.51, R^2^ = 0.182, *p* = 0.001, Table 3). Identification effort (i.e., number of specimens identified per sample) also had a significant effect on thrips assemblages (F = 30.51, R^2^ = 0.062, *p* = 0.001, Table 3), although it explained a smaller proportion of the variation than the sample method. Differences in multivariate dispersion among methods were also significant (PERMDISP, F = 4.58, *p* = 0.0013, Table 4), indicating that sampling methods varied in both species composition and within-group variability. In addition, non-metric multidimensional scaling (NMDS) provided a reliable representation of these differences with a stress value of 0.097 (Figure 4), showing partial separation among sampling methods with some overlap, which is consistent with the PERMANOVA results.

*Frankliniella bispinosa* (Morgan), the Florida flower thrips, was the most abundant species in whole plant samples, representing 77% of identified specimens (Table 2). In addition, *F. bispinosa* was consistently captured across pan trap colors in both research and commercial sites and was the most abundant species in most pan traps, accounting for 67.8%, 76.4%, and 79.2% of the identified specimens in yellow pan traps and blue pan traps in research, and blue pan traps in commercial fields, respectively (Table 2). In yellow pan traps in commercial fields, *Microcephalothrips abdominalis* (Crawford), the composite thrips, was the dominant species and accounted for 54.2% of identified specimens, and *F. bispinosa* was the second most abundant species (38.6%) (Table 2).

*Leucothrips piercei* (Morgan) (11.6%) and *Frankiniella fusca* (Hinds) (6%) were the second and third most abundant species in whole plant samples, whereas only a few individuals (0 to 2) of these species were observed from pan trap samples. Additionally, *Scirtothrips dorsalis* Hood, the chilli thrips, was only captured in lettuce samples and not in pan traps (Table 2). In contrast, *Frankliniella insularis* Franklin, *Megalurothrips usitatus* (Bagnall), and *Thrips orientalis* (Bagnall) were only captured by pan traps but not observed in lettuce plant samples (Table 2).

Thrips species known to transmit orthotospovirus, including *F. occidentalis, F. fusca*, and *Frankliniella schultzei* Trybom were present in low numbers in both plant samples and pan traps (Table 2). These species represented 0.66%, 6%, and 0.99%, respectively, of the total identified specimens in plant samples, and 1.07%, 0.23%, and 2.15%, respectively, of the total identified specimens captured from pan traps across colors and sites (Table 2).

## 4. Discussion

Five sampling methods—whole plant sampling, blue pan trap, yellow pan trap, blue sticky trap, and yellow sticky trap—were used in this study to evaluate their efficacy for thrips monitoring. A total of 9814 thrips were collected across all samples. Weekly thrips abundance showed that the numbers of thrips captured varies by the sampling method used, highlighting the importance of using optimal scouting methods, as it may result in different outcomes and potentially affect important management decisions. However, similar population dynamic patterns were observed among methods, with comparable timing of pick thrips captured (Figure 2 and Figure 3). Of 9814 thrips collected, 3883 specimens (approximately 40%) were identified to species or genus levels, this excluded 22 specimens belonging to the suborder Tubulifera (Table 2).

Whole plant sampling captured the lowest number of thrips among all sampling methods evaluated. This may be because, unlike the sticky traps or pan traps, which were deployed for a one-week period to capture insects over time, whole plant sampling captures only insects present at the moment of sampling. Although the number of collected thrips from plant samples were the lowest, the thrips captured using this method represent species that are likely to feed on lettuce, rather than incidental bycatch. However, whole plant sampling is a destructive method requiring the removal of lettuce plants, which translate into product loss in commercial fields. Additionally, it was the most labor-intensive and time-consuming method in terms of post-sampling processing, as lettuce leaves need to be cut and repeatedly filtered to ensure that no thrips hiding between leaves are missing.

Sticky traps, especially blue colored ones, captured significantly more thrips than whole plant samples or pan traps in some weeks (Figure 2 and Figure 3). A sticky trap is an effective tool and widely used across diverse environmental conditions to monitor various insect species [33,34,35,36]. However, glue adhesive from the traps made specimen removal difficult and week-long environmental exposure in the fields degraded specimen quality, making species identification extremely challenging. Sticky traps can still be a useful monitoring tool for larger or small insects with well-documented species composition in a region, particularly when the dominant species is the target of monitoring efforts. Additionally, due to their high capture efficacy to collect large number of thrips, sticky traps could be placed along field edges for surveillance to monitor thrips dispersal and activity during the early growing season [37].

In contrast to sticky traps, pan traps preserved specimens well enough for identification even after one week in the fields. Considering the number of thrips collected using pan traps, this method offered a more practical option than sticky traps, as both number of individuals and species identification were important components of this study. While pan traps were less labor intensive to collect and faster to process than whole plant samples, they are typically left unattended for extended periods. As a result, environmental factors, such as soil debris caused by strong winds, overflow from heavy rains, and destruction from farm equipment, can negatively affect pan trap collections. It is important to note that both sticky traps and pan traps capture a general sample of species diversity present in the surrounding area and may not represent the thrips species that are actively feeding on the lettuce.

No consistent significant differences were observed in the number of thrips collected between yellow and blue sticky traps and pan traps in our study (Figure 2 and Figure 3). In contrast, numerous studies have reported significant effects of trap color on thrips sampling efficiency. For instance, multiple studies have shown that blue sticky traps capture more thrips than yellow traps, particularly for *F. occidentalis* [38,39,40,41]. Johansen et al. compared blue and yellow sticky traps equipped with blue light-emitting diodes and found that blue traps were more attractive to *F. occidentalis* than yellow traps in protected herb production [42]. Similarly, higher numbers of *F. occidentalis* and *Thrips tabaci* Lindeman captured on blue than yellow sticky traps were observed in lettuce [37]. More recently, Nkafu et al. demonstrated that thrips color preference is species-specific and can be influenced by additional factors, including crop type, temperature, and relative humidity [43].

*Frankliniella bispinosa* was the most abundant thrips species across all sampling methods (Table 2). This species is commonly known as the Florida flower thrips, found in many crops and wild plants in Florida, and it is endemic to Florida and Georgia [44]. While it is not considered a pest in lettuce, *F. bispinosa* is a pest in several crops in Florida, including bell pepper [45]. Additionally, *F. bispinosa* has been shown to transmit tomato spotted wilt virus (TSWV) from pepper to pepper under laboratory conditions, with a transmission rate of 50% [46]. Tomato spotted wilt virus is among the most destructive orthotospoviruses infecting lettuce [10,47,48,49,50]. However, Funderburk suggested that *F. bispinosa* may not be a competent vector of TSWV under natural environments, based on the absence of TSWV epidemics in central and southern Florida despite the presence of a high population of *F. bispinosa* [51]. Consequently, the vector competence of *F. bispinosa* in field conditions remains unknown. Given the prevalence of TSWV in many crops in southern Florida [52], the high populations of *F. bispinosas* warrant close monitoring, and their uncertain vector competence also requires further investigation.

Another important role of *F. bispinosa* is that it serves as an effective competitor of *F. occidentalis* [51]. Shifts in species dominance from *F. bispinosa* (a less damaging species) to *F. occidentalis* (a highly destructive pest) have been reported owing to differences in insecticide susceptibly, with *F. bispinosa* being highly susceptible and *F. occidentalis* being resistant to some insecticides [14,51]. Our observation of *F. bispinosa* as the dominant species is consistent with the minimal use of insecticides in commercial lettuce farms in the EAA, where thrips are not major management concerns and insecticide applications are therefore limited (anonymous lettuce farm manager, personal communication).

*Leucothrips piercei* was the second most abundant thrips found in whole plant samples, but not in pan trap samples (Table 2). *Lecuothrips piercei* has been mostly documented in Thysanoptera surveys. For example, it was detected in citrus in Florida [1], in redbud trees in Illinois [53], and in common beans (*Phaseolus vulgaris*) in Japan [54], without apparent damage to those plants. However, *L. piercei* was reported as a major pest of cotton in Peru [55] and in pepper in Argentina [56]. While no visible damage to lettuce was detected, the high population level of *L. piercei* observed in the EAA suggests that this species should be closely monitored.

*Microcephalothrips abdominalis* was the most abundant species (accounting for approximately 54% of thrips identified) in yellow pan traps in commercial farms and was the second most abundant species in other pan traps (Table 2). *Microcephalothrips abdominalis* is an effective pollinator [57] and has been identified as a vector of the tobacco streak virus (TSV) [58,59], which can be transmitted by thrips, pollen, and seed. This virus has a wide host range, infecting more than 200 plant species, including lettuce [60]. Tobacco streak virus has been present in beans in Florida since 1994 [61] and has recently emerged as a growing problem in southern Florida [62,63]. Therefore, *M. abdominalis* should also be closely monitored in the area.

Vectors of INSV and TWSV, including *F. fusca*, *F. occidentalis* and *F. schultzei,* [3,4], were detected in relatively low numbers in both research and commercial lettuce fields in this study (Table 2). *Frankliniella fusca* was primarily detected in whole plant samples, with minimal representation in pan traps. *Frankliniella occidentalis,* although consistently present in all trap types, observed at even lower number (Table 2). These findings contrast with observations from the major lettuce producing region of Salinas, California, where *F. occidentalis* comprised 84–98% of thrips collected from lettuce and was identified as the predominant species [10,64]. The comparatively low prevalence of these vector species in the EAA is advantageous for limiting the spread of INSV into lettuce fields, despite the virus occurring in other crops within the region. Nonetheless, the detection of these vector species is noteworthy and underscores the need for continued surveillance to better understand their phenology. In addition, robust lettuce sampling and screening for INSV and other orthotospovirus are needed to more clearly define their epidemiological status in the area.

It is important to note that limited insecticides were applied in the commercial fields (anonymous lettuce farm manager, personal communication). Nonetheless, other pesticides, such as fungicides, or other agricultural practices and environmental conditions in commercial fields may have influenced thrips abundance and species composition. Therefore, differences between research and commercial fields should be interpreted with caution, as they may reflect pesticides effects or other environmental factors in addition to sampling method.

Additionally, seasonal differences in environmental conditions, such as temperature and precipitation, may influence thrips abundance. However, the effects of these factors were not evaluated in this study. Future research incorporating environmental data would help clarify the role of these factors in shaping thrips population dynamics.

While the observed differences in species composition provide insight into method-specific species detection (Table 3 and Figure 4), interpretation of these results need to carefully take methodological constraints into consideration. In our analysis of species composition, the number of identified specimens varied among sampling methods due to practical constraints on the number of individuals processed per sample, particularly for pan traps. Therefore, species composition estimates may be biased toward more abundant species in methods with lower identification rate, and rare species may be underrepresented. Although we accounted for this by including the number of identified specimens per sample as a covariate in multivariate analyses, caution is warranted when interpreting differences in species composition among methods. Some observed differences may partly reflect variations in subsampling intensity rather than true ecological differences. In addition, PERMDISP indicated significant heterogeneity in multivariate dispersion, suggesting that differences among methods reflect both shifts in species composition and variation in within-method variability. Therefore, PERMANOVA results should be interpreted with caution. Nevertheless, NMDS patterns and the relatively large effect of the sampling method indicate that methodological differences remain an important driver of observed assemblage structure. Future studies could address this limitation by increasing identification rates or applying rarefaction or other standardization approaches.

## 5. Conclusions

As part of proactive management efforts to safeguard Florida’s major lettuce producing region from thrips and thrips-transmitted viruses, thrips population abundance and species composition were assessed in this study. Although peak thrips abundance time detected using whole plant samples, pan traps, and sticky traps showed similar trends, it was concluded that pan traps were the most efficient and practical method for survey thrips in lettuce fields due to the high number of individuals collected and the preservation of specimens for species identification. No consistent significant differences were observed in number of thrips collected from yellow and blue pan traps. A total of at least eleven thrips species were identified, with *F. bispinosa* being the dominant species, followed by *M. abdominalis* and *L. piercei*; together, these species comprised approximately 89% of the identified thrips. The presence of important orthotospoviruses vectors, *F. fusca*, *F. occidentalis*, and *F. schultzei*, was also confirmed, highlighting the importance of thrips surveillance for lettuce crop protection.

## Figures and Tables

**Table 1:** Analysis of deviance table of the generalized linear mixed-effects models to compare the number of thrips affected by sampling method, plant age, and the interaction between sampling method and plant age in research and commercial fields.

Fields	Effects	Chisq	df	*p* Value
R1	Sampling method	890.07	4	<2.2 × 10^−16^
	Plant age	149.47	3	<2.2 × 10^−16^
	Sampling method × plant age	104.92	12	<2.2 × 10^−16^
R2	Sampling method	1746.13	4	<2.2 × 10^−16^
	Plant age	611.87	6	<2.2 × 10^−16^
	Sampling method × plant age	153.69	24	<2.2 × 10^−16^
C1	Sampling method	13.1564	3	0.0043104
	Plant age	20.3844	3	0.0001413
	Sampling method × plant age	6.4381	9	0.6954
C2	Sampling method	32.78	3	3.58 × 10^−7^
	Plant age	213.64	2	<2.2 × 10^−16^
	Sampling method × plant age	8.91	6	0.1787
C3	Sampling method	106.137	3	<2.2 × 10^−16^
	Plant age	57.032	3	2.53 × 10^−12^
	Sampling method × plant age	10.429	9	0.3169
C4	Sampling method	39.37	3	1.45 × 10^−8^
	Plant age	347.88	3	<2.2 × 10^−16^
	Sampling method × plant age	35.82	9	4.26 × 10^−5^

**Table 2:** Thrips species composition by sampling method. Values represent the proportion (%) of total identified specimens, and the numbers in parentheses indicate the number of specimens collected (*n*).

Scientific Name	Common Name	Whole Plant Sample	Yellow Pan trap (Research) *	Blue Pan Trap (Research) *	Yellow Pan Trap (Commercial) *	Blue Pan Trap (Commercial) *
*Caliothrips phaseoli*	South American bean thrips	1.23% (26)	0.25% (1)	0	0.25% (1)	0.37% (2)
*Echinothrips americanus*	Poinsettia thrips, impatiens thrips	0.19% (4)	0	0	0.25% (1)	0
*Frankliniella bispinosa*	Florida flower thrips	77.05% (1628)	67.81% (276)	76.44% (318)	38.56% (155)	79.82% (435)
*Frankliniella fusca*	Tabacco thrips	6.01% (127)	0	0.48% (2)	0.25% (1)	0.18% (1)
*Frankliniella insularis*	-	0	2.21% (9)	1.20% (5)	1.24% (5)	1.28% (7)
*Frankliniella occidentalis*	Western flower thrips	0.66% (14)	1.97% (8)	0.48% (2)	1.24% (5)	0.73% (4)
*Frankliniella schultzei*	Common blossom thrips	0.99% (21)	1.23% (5)	3.13% (13)	1.99% (8)	2.20% (12)
*Fulmekiola serrata*	Sugarcane thrips	0.05% (1)	0.74% (3)	0	0	0.37% (2)
*Leucothrips piercei*	-	11.59% (245)	0	0.24% (1)	0	0
*Megalurothrips usitatus*	Twice-banded bean thrips	0	0	0.48% (2)	0.50% (2)	2.94% (16)
*Microcephalothrips abdominalis*	Composite thrips	0.71% (15)	23.10% (94)	8.17% (34)	54.23% (218)	6.79% (37)
*Pseudothrips inequalis*	-	0.09% (2)	0	0	0.25% (1)	0.18% (1)
*Scirtothrips dorsalis*	Chilli thrips	0.38% (8)	0	0	0	0
*Thrips orientalis*	Star jasmine thrips	0	0	0.48% (2)	0.25% (1)	0
*Thrips palmi*	Melon thrips	0.09% (2)	0.25% (1)	2.64% (11)	0	1.65% (9)
*Thrips parvispinus*	Short-spined thrips	0.19% (4)	0.25% (1)	0.96% (4)	0	0.92% (5)
*Anaphothrips* sp.	-	0.57% (12)	0	1.44% (6)	0.25% (1)	0.73% (4)
*Arorothrips* sp.	-	0.09% (2)	0	0.24% (1)	0	0.55% (3)
*Chirothrips* sp.	-	0	0.25% (1)	0	0	0
*Stenchaetothrips* sp.	-	0.14% (3)	0.49% (2)	2.88% (12)	0.50% (2)	0.73% (4)
*Neohydatothrips* sp.	-	0	0.49% (2)	0.24% (1)	0.25% (1)	0.55% (3)
Suborder Tubulifera	-	0.38% (8)	0.98% (4)	0.48% (2)	0.50% (2)	1.10% (6)
Thrips identified		84.49% (2114)	17.91% (407)	13.29% (416)	61.85% (402)	43.32% (545)
Thrips collected		2502	2273	3131	650	1258

* A maximum of 15 specimens from each sample were identified.

**Table 3:** Results of permutational multivariate analysis of variance (PERMANOVA) testing the effects of sampling method and identification effort on thrips community composition based on Bray–Curtis dissimilarities (999 permutations).

Effects	df	Sum Sq	*R^2^*	F Value	*p* Value
Sampling method	4	16.692	0.182	22.51	0.001
Identification effort	1	5.656	0.062	30.51	0.001
Residual	374	69.332	0.756		
Total	379	91.680	1.000		

**Table 4:** Analysis of homogeneity of multivariate dispersion (PERMDISP) testing for differences in variability of thrips assemblages among sampling methods.

	df	Sum Sq	Mean Sq	F Value	*p* Value
Sampling method	4	1.299	0.325	4.58	0.0013
Residual	375	26.591	0.071		

## Data Availability

The raw data supporting the conclusions of this article will be made available by the authors on request.
